# Diagnostic accuracy of a pocket screening spirometer in diagnosing chronic obstructive pulmonary disease in general practice: a cross sectional validation study using tertiary care as a reference

**DOI:** 10.1186/s12875-016-0518-8

**Published:** 2016-08-19

**Authors:** Marina Labor, Žarko Vrbica, Ivan Gudelj, Slavica Labor, Davor Plavec

**Affiliations:** 1 Department of Pulmonology, University Hospital Center Osijek, Josipa Huttlera 4, Osijek, Croatia; 2 Faculty of Medicine, J.J. Strossmayer University of Osijek, Ulica cara Hadrijana 10E, Osijek, Croatia; 3 Department of Pulmonology an Immunology, General Hospital Dubrovnik, Dr. Roka Mišetića 2, Dubrovnik, Croatia; 4 University of Dubrovnik, Branitelja Dubrovnika 29, Dubrovnik, Croatia; 5 Department of Pulmonology, University Hospital Center Split, Spinčićeva 1, Split, Croatia; 6 Research Department, Children’s Hospital Srebrnjak, Srebrnjak 100, Zagreb, Croatia

**Keywords:** COPD, Diagnosis, General practice, Screening, Sensitivity and specificity

## Abstract

**Background:**

COPD-6™ is a lung function testing device for a rapid pre-spirometry testing to screen-out at-risk individuals not having COPD and indicating those at risk. The aim of this study was to validate COPD-6™ lung function testing (index test) in general practice in discriminating patients with COPD out of the population at risk - smokers/ex-smokers with no previous diagnosis of COPD, using measurements at tertiary care as reference standard.

**Methods:**

Consecutive 227 subjects (115 women, 185 smokers/42 ex-smokers, ≥20 pack-years) with no previous diagnosis of COPD, aged 52.5 (SD 6.8) years from 26 general practitioners (GPs) were recruited, lung function tested with COPD-6™, referred to the tertiary institution for repeated COPD-6™ testing followed by spirometry with a bronchodilator (salbutamol), examination, and pulmonologist consultation for the diagnosis and severity of COPD.

**Results:**

COPD was diagnosed in 43 subjects (18.9 %), with an AUC of 0.827 (95 % CI 0.769-0.875, *P* < 0.001) for the diagnosis of COPD when lung function was measured using COPD-6™ in GP’s office with a specificity of 100 % (95 % CI, 97.95–100 %) but a very low sensitivity of 32.56 % (95 % CI, 20.49–47.48 %). Significant agreement for forced expiratory volume in 1 s measured at GP’s office and at lung function lab was found (mean difference 0.01 L, *p* = 0.667) but not for other measured parameters (*p* < 0.001 for all).

**Conclusions:**

Our study results point out that active case finding in a population at risk for COPD should be instituted (almost 20 % of undiagnosed COPD). Based on our results lung function testing with COPD-6™ can substitute spirometry testing in cases where it is not readily available to the patient/physician taken into account that the traditional FEV_1_/FEV_6_ cutoff value of <0.7 is not the only criterion for diagnosis and/or further referral.

**Trial registration:**

ClinicalTrials.gov Identifier NCT01550679 Registered 28 September 2014, retrospectively registered

## Background

COPD is one of the leading causes of morbidity and mortality worldwide [1]. Existing prevalence data show variations due to survey methods reflecting also a widespread under-diagnosis of COPD, even in patients with developed respiratory symptoms [2–5]. On the other hand patients with a mild COPD already have substantial reduction in all parameters of health related QoL (HRQoL) [6]. Modern strategies for COPD management are stressing the importance of primary care physician’s office-based assessments of patients at risk, thus significantly increasing the number of timely diagnosed COPD patients [7].

Spirometry with the documented post-bronchodilator FEV_1_/FVC <0.70 is required to make a diagnosis of COPD in a clinical context of the disease [1]. Spirometry in general practitioners’ (GPs) office would be adequate to make a diagnosis, but there are many obstacles to that strategy; the price of equipment, reimbursement strategies, quality of spirometry in such an environment, insufficient training, and experience in testing [8]. Unavailability of a spirometry often leads to over-diagnosis of COPD, made only based on symptoms and exposure data without confirmatory lung function testing, leading to overtreatment and negative impact on morbidity and mortality [9–11].

COPD-6™ (4000 COPD-6™ Respiratory Monitor, Vitalograph Ltd., Buckingham, UK) is a cheap, simple lung function testing device approved as a rapid pre-spirometry testing tool to screen-out the at-risk individuals who do not have COPD and indicate those that may be at risk. It is a simple device, easy to learn how to operate, having the readout without the risk of false COPD negatives, thus focusing spirometry resources on a smaller population with most of the risk. Four major problems could arise from using COPD-6™ as a screening device instead of spirometry: (1) using FEV_6_ instead of FVC could underestimate the later; (2) no post-bronchodilator testing; (3) no flow-volume curve presentation; (4) a single criterion for fixed airflow limitation as defined in GOLD initiative (FEV_1_/FEV_6_ < 0.70) thus possibly producing a significant over-diagnosis in elderly (>70 years of age) according to The Global Lung Function Initiative data [12].

Having all that in mind, we wanted to explore the diagnostic accuracy of COPD-6™ in a population of smokers/ex-smokers with a significant exposure to cigarette smoke, with no previous diagnosis of COPD, in a general practice setting comparing it to the ‘gold standard’, spirometry conducted in a lung function laboratory at the tertiary care level (university and teaching hospitals) by experienced staff with special training. This is a population with expected 25 % of undiagnosed COPD cases in which such case identification is recommended [1]. So, the aim of this study was to validate COPD-6™ lung function testing (index test) in general practice in discriminating patients with COPD out of the population at risk - smokers/ex-smokers with no previous diagnosis of COPD, using measurements at tertiary care as reference standard. The secondary goal was to assess the agreement between lung function measurements between methods (COPD-6™ vs. spirometry) and between health care settings (primary vs. tertiary care).

## Methods

### Study framework

This prospective cohort study was a part of broader research project (Early detection of COPD patients in GOLD 0 (smokers) population – MARKO project). The whole protocol of the MARKO project can be found at https://clinicaltrials.gov/ct2/show/NCT01550679. The study was approved by the local ethics committee and conducted according to the Declaration of Helsinki and other relevant international and national laws. The patients were approached by their GPs during any (unrelated to respiratory problems) visit to their office if they were smokers or ex-smokers of the predefined age group for the study together with the prescreening for inclusion/exclusion criteria using a structured interview. Eligible patients were given the Informed consent document with enough time to read it and to discuss any relevant issues regarding the study before they signed the written consent. They were informed about the prospective nature of the study and their right to withdraw their consent and claim the withdrawal of all gathered data and destroying all biological samples at any time without any explanation, obligation or consequence from their side. All participants signed the written consent before starting any procedure for the study.

### Subjects

The consecutive patients from 26 GPs (representing the same number of GP offices) were recruited based on inclusion/exclusion criteria. We decided on consecutive patients based on the limited number of insured persons under the care by each GP (approx. 1700), and relatively low response rate for a public health campaigns in our country. Inclusion criteria were: written consent; smokers/ex-smokers of either sex, aged 40–65 years with a smoking history of at least 20 pack-years (calculated as number of cigarettes smoked per day multiplied by the number of years of smoking divided by 20); with no previous diagnosis of COPD. Exclusion criteria were: any clinically relevant chronic disease significantly affecting QoL at the time of the first visit (cardiovascular, cerebrovascular, diabetes, hepatitis, nephropathy, chronic dialysis, systemic disorder, cancer); immunosuppressive therapy; preceding acute respiratory disease 4 weeks before the visit; hospitalization for any reason during past 3 months; myocardial infarction, cerebrovascular infarction or transient ischemic attack during past 6 months; diagnosis of asthma; and an inability to perform the diagnostic protocol. Exclusion criteria were introduced not to exclude patients having comorbidities common in COPD, but to exclude the ones that represent acute/subacute clinical states/disorders or states of recovery from major clinical disorders representing an absolute or relative contraindication for spirometry or significantly influencing the diagnostic process or an already present diagnosis of respiratory disorder (e.g., asthma).

### Study workup

All GPs went through short small groups training and were provided with the COPD-6™ devices. GPs were not extensively trained in spirometry or assessed for their skill level because we wanted that the measurements would be performed as close as possible to the regular real-life clinical situation where GPs scarcely use these measurements. After the examination at the GP’s office and lung function testing with COPD-6™ (index test), patients were referred after 2–4 weeks to one of the tertiary institutions to a designated team consisting of a pulmonologist, research nurse and lung function laboratory technician. Standard diagnostic workup consisted of repeated COPD-6™ lung function testing followed by spirometry with a bronchodilator (salbutamol), history, physical examination and specialist consultation. Patients with no previous diagnosis of COPD were chosen to avoid bias coming from a previous knowledge of a diagnosis but allowing a significant subsample of subject with COPD (according to previous studies up to 25 % of subjects in this population has an undiagnosed COPD) [1]. To avoid the second possible bias, the team at the tertiary care institution was blinded for the results of the COPD-6™ measurements performed at GP’s office. After the workup conducted at the tertiary care institution the pulmonologist made the diagnosis and severity assessment of COPD according to GOLD: relevant exposure, respiratory symptoms characteristic for COPD and fixed airflow limitation (post bronchodilator FEV_1_/FVC <0.70) [1]. This was used as a reference standard for this study.

### Lung function measurements

Lung function measurements using COPD-6™ were performed according to the manufacturer’s recommendations and ATS/ERS guidelines [11]. Measurements were repeated until 3 technically satisfactory efforts were performed. COPD-6™ has a quality assessment built in the device and marks the technically inadequate measurement with the exclamation mark. Exclamation mark appears when the time of expiration is too short or coughing during expiration was present. After 3 technically satisfactory efforts the device automatically choses the best one and these results were recorded as absolute values for forced expiratory volume in 1 s (FEV_1_ in L), forced expiratory volume in 6 s (FEV_6_ in L), FEV_1_/FEV_6_ ratio (%), and lung age (years) and as % of predicted values for FEV_1_, FEV_6_ and FEV_1_/FEV_6_ according to prediction equations already in the device calculated according to sex, age and height. The device uses the pre-specified cut-off levels for visually suggesting the preliminary diagnosis of COPD (FEV_1_/FEV_6_ ratio of <0.7) and assesses the severity according to GOLD initiative [1], so we used this criterion as a positive index test for further comparisons. The same procedure was followed at both sites (GP’s offices and lung function labs in a tertiary care hospitals).

Spirometry was performed using computerized pneumotachographs (Jaeger®, CareFusion, CA, USA) using the same procedure at all clinical sites (lung function labs at tertiary hospitals) according to ATS/ERS guidelines [13]. The best of three technically satisfactory efforts was recorded. Bronchodilator test was performed by repeated spirometry 20 min after the inhalation of 400 mcg of salbutamol using the inhalation chamber. Absolute values of FEV_1_, forced expiratory capacity (FVC), FEV_1_/FVC ratio were together with sex, age and height entered into the Excel sheet (Microsoft® Excel® 2013, Microsoft Corporation, USA) for all subjects and using predicted values equation from Quanjer, % of predicted was calculated in a single act using Excel [14]. Tertiary care postbronchodilator spirometry measurements at lung function laboratory were used as reference standard for this study.

### Data analyses

Data analysis was performed using STATISTICA version 12 (StatSoft, Inc., OK, USA) and MedCalc Statistical Software version 15.8 (MedCalc Software bvba, Ostend, Belgium; https://www.medcalc.org; 2015). Minimal sample size of 70 subjects (14 positive and 56 negative) was calculated for the expected area under the curve (AUC) of 0.8 with a statistical power of 95 % (beta 0.05) and alpha of 0.05. Categorical data was presented as absolute and relative (%) numbers. Continuous variables were presented as mean and standard deviations (SD). Categorical data was compared between subgroups using chi-square (*χ*2) test and continuous variables using Student’s *t*-test or Mann-Whitney *U* test and analysis of variance (ANOVA). The criteria to use Student’s *t*-test and ANOVA were checked and fulfilled before the tests were performed. Agreement between lung function measurement methods was conducted using Bland-Altman statistics and plots. Utility of FEV_1_/FEV_6_ measured using COPD-6™ at GP’s office (index test) for diagnosing COPD was analyzed comparing it to the reference standard using receiver operator curve (ROC) analysis and data was presented as AUC together with sensitivity, specificity and positive and negative predictive values together with 95 % confidence intervals (CIs). *P* < 0.05 was used as statistically significant for all analyses.

## Results

**Fig. 1:**
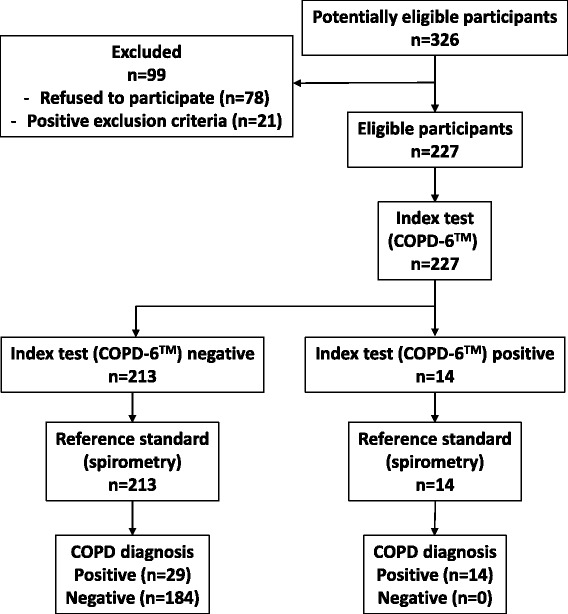
Diagram of flow of patients through the study

Out of 326 consecutive prescreened smokers of eligible age and smoking history, 227 (69.6 %) subjects (115 women) at risk for COPD (185 smokers and 42 ex-smokers) aged 52.5 (SD 6.8) years were included in this study (78 refused to participate and 21 were excluded based on exclusion criteria, Fig. 1). The basic demographic data for included subjects are displayed in Table 1. The diagnosis of COPD (reference diagnosis) was made in 43 (18.9 %) subjects with no significant difference between men and women (*χ*2 = 2.711, *P* = 0.100) or between smokers and ex-smokers (*χ*2 = 1.763, *P* = 0.185). Cross-tabulation of an index test positivity against the reference standard is presented in Table 2. No significant difference was found between subjects with COPD and no-COPD for age (*t* = 1.139, *P* = 0.256), presence of comorbid disorders (55.5 %) and chronic treatment (*χ*2 = 0.049, *P* = 0.825; *χ*2 = 0.125, *P* = 0.724; respectively), BMI (*t* = 0.100, *P* = 0.921) or smoking habit (*p* > 0.100 for all parameters of smoking habit). Also no clustering of COPD diagnosis, demographics or smoking habit data was evident for different GPs (*p* > 0.100 for all comparisons). In Table 3 lung function data is presented according to the existence and severity of COPD (24, 10.4 % GOLD stage 1 and 19, 8.2 % GOLD stage 2).

**Table 1:** Demographics, smoking habit, presence of comorbid disorders and chronic treatment other than that for COPD according to final COPD diagnosis (*N* = 227)

Variables	All (*N* = 227)	COPD (*n* = 43)	Non-COPD (*n* = 184)	Statistics
Women (%)	115 (50.7)	17 (39.5)	99 (53.2)	*χ*2 = 2.711, *P* = 0.100
Age (years), mean ± SD	52.5 ± 6.8	53.6 ± 7.0	52.3 ± 6.7	*t* = 1.139, *P* = 0.256
BMI (kgm^−2^), mean ± SD	26.5 ± 4.2	26.5 ± 5.2	26.5 ± 3.9	*t* = 0.100, *P* = 0.921
Active smokers (%)	185 (84.9)	32 (84.2)	153 (85.0)	*χ*2 = 0.015, *P* = 0.902
Years of smoking, mean ± SD	30.6 ± 6.9	32.0 ± 6.4	30.3 ± 6.9	z = 1.641, *P* = 0.101
Cigarettes/day, mean ± SD	24.6 ± 9.1	24.4 ± 8.0	24.6 ± 9.2	z = 0.241, *p* = 0.809
Pack-years, mean ± SD	37.9 ± 17.4	39.1 ± 14.3	37.5 ± 17.5	z = 1.310, *p* = 0.190
Presence of comorbid disorders (%)	126 (55.5)	22 (51.2)	104 (56.5)	*χ*2 = 0.049, *P* = 0.825
Chronic treatment (%)	99 (43.6)	16 (37.2)	83 (45.1)	*χ*2 = 0.125, *P* = 0.724

*χ2* chi-square test results, *t* result of Student’s *t*-test, *z* result of Mann-Whitney *U* test, *SD* standard deviation, *BMI* body mass index calculated as the ratio of body weight in kg and squared body height in meters

**Table 2:** Cross-tabulation of the results of index test (COPD-6™) against the reference standard (spirometry) (*N* = 227)

Index test (COPD-6™)	Reference standard (spirometry)		
	Positive	Negative	Total
Positive Negative	14 29	0 184	14 213
Total	43	184	227

**Table 3:** Lung function (COPD-6™, spirometry) according to the presence and severity of COPD according to GOLD stages

	Lung function	All	Non-COPD	COPD GOLD 1	COPD GOLD 2	Statistics
		(*N* = 227)	(*n* = 184)	(*n* = 24)	(*n* = 19)	
COPD-6™	FEV_1_ (% predicted)	94.3 ± 15.6	97.6 ± 13.3	90.9 ± 13.0	67.5 ± 12.2	*F* = 46.27 *P* < 0.001
	FEV_6_ (% predicted)	93.9 ± 16.2	96.0 ± 15.3	94.7 ± 14.9	74.5 ± 13.7	*F* = 17.27 *P* < 0.001
	FEV_1_/FEV_6_ (%)	0.845 ± 0.085	0.864 ± 0.071	0.781 ± 0.083	0.757 ± 0.117	*F* = 25.84 *P* < 0.001
	Lung age (yrs)	60.7 ± 13.9	57.7 ± 11.1	64.0 ± 11.1	84.6 ± 15.7	*F* = 48.07 *P* < 0.001
Spirometry	FEV_1_ (% predicted)	97.9 ± 15.3	101.5 ± 12.9	92.9 ± 10.2	71.1 ± 12.7	*F* = 51.50 *P* < 0.001
	FVC (% predicted)	109.3 ± 17.0	110.8 ± 16.7	112.7 ± 11.8	91.1 ± 16.2	*F* = 13.29 *P* < 0.001
	FEV_1_/FVC (%)	0.742 ± 0.073	0.761 ± 0.060	0.665 ± 0.055	0.650 ± 0.073	*F* = 49.14 *P* < 0.001
	ΔFEV_1_ (%)	1.39 ± 4.00	1.40 ± 3.79	2.92 ± 4.25	−0.93 ± 7.68	*F* = 3.011 *P* = 0.051

Data for all variables is presented as mean ± standard deviation; *FEV*_*1*_ forced expiratory volume in 1 s, *FEV*_*6*_ forced expiratory volume in 6 s, *FVC* forced expiratory volume, *ΔFEV*_*1*_ post-bronchodilator change in FEV_1_ (measured 20 min after inhalation of 400 μg of salbutamol), F – result of ANOVA for between group comparisons

**Fig. 2:**
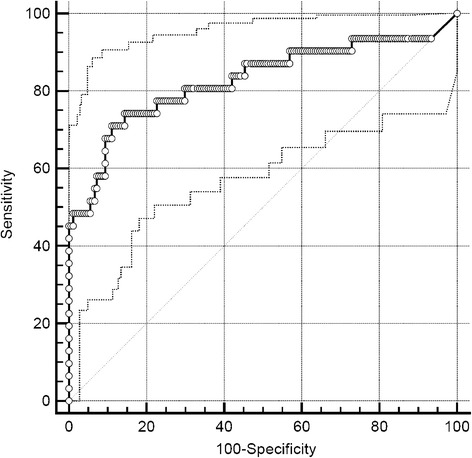
ROC curve for the diagnosis of COPD using COPD-6™ at the GP’s office. ROC curve plot (AUC 0.827, 95 % CI 0.769–0.875, *P* < 0.001) was based on FEV_1_/FEV_6_ measurements using COPD-6™ at the GP’s office using COPD diagnosis made by pulmonologist at tertiary care hospital as criterion variable; dotted lines represent 95 % confidence intervals

ROC curve analyses of FEV_1_/FEV_6_ measurements for the diagnosis of COPD using COPD-6™ at the GPs office and at the lung function lab at the tertiary care hospital gave an AUC of 0.827 (95 % CI 0.769–0.875, *P* < 0.001; Fig. 2) and 0.849 (95 % CI 0.788–0.898, *P* < 0.001) being significantly different to spirometry (AUC 0.961, 95 % CI 0.920–0.984, z statistic = 2.501, *P* = 0.012; z statistic = 4.058, *P* < 0.001; respectively). Using the usual (pre-specified) threshold of <0.7 for FEV_1_/FEV_6_ for the diagnosis of COPD for the lung function (COPD-6™) values measured at GP’s offices gave the highest specificity of 100 % (95 % CI, 97.95–100 %) but a very low sensitivity of 32.56 % (95 % CI, 20.49–47.48 %) with a PPV of 100 % (95 % CI, 78.47–100 %) and NPV of 86.38 % (95 % CI, 81.13–90.35 %).

Exploratory analyses using the change in threshold gave somewhat better overall results with a change of threshold to ≤0.78 for FEV_1_/FEV_6_ that gave a specificity of 88.95 % (95 % CI, 83.83–92.60 %) with a sensitivity of 70.97 % (95 % CI, 54.72–85.03 %), a PPV of 52.38 % (95 % CI, 37.72–66.64 %), NPV of 94.71 % (95 % CI, 89.90–97.76 %). The highest NPV (95.74 %, 95 % CI 90.93–98.79 %) was achieved with a cut-off value of 0.85 with the negative likelihood value of 0.26 (95 % CI, 0.20–0.33).

**Fig. 3:**
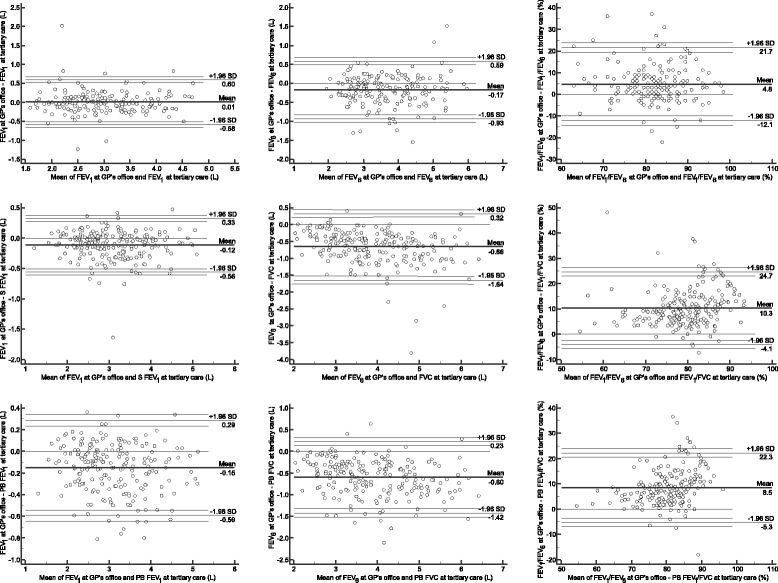
Bland-Altman plots for between methods and points of care comparisons for lung function parameters. Bland-Altman plots are presented as plots of difference between two measurements [y-axis] plotted against the mean of two measurements [x-axis]; graphs at columns represents parameters measured using COPD-6™ at the GP’s office: first column – FEV_1_, second column – FEV_6_, third column – FEV_1_/FEV_6_; rows represent mean of two measurements: first row – COPD-6™ at tertiary care, second row – spirometry (S), third row – postbronhodilator (PB) spirometry

Methods comparison between lung function values measured at GP’s offices and values measured at lung function labs at tertiary care hospitals are shown in Table 4 and Fig. 3. FEV_1_ values measured using COPD-6™ at GP’s offices showed small differences with the same measurement at tertiary care and no clinically relevant differences when compared with spirometric measurement and postbronchodilator one (mean difference, −0.12 L and −0.16 L, *P* < 0.001 for both, Bland-Altman statistics; Table 4 and Fig. 3). Point of care comparison for FEV_6_ (primary vs. tertiary care) showed clinically non-relevant difference (Table 4 and Fig. 3) but method comparison with spirometric and postbronchodilator measurements of FVC showed significant and clinically relevant differences (mean difference, −0.66 L and −0.60 L, *P* < 0.001 for both, Bland-Altman statistics) when compared to reference measures with a trend of increasing the difference with larger values (Table 4 and Fig. 3). Comparison of FEV_1_/FEV_6_ values measured using COPD-6™ showed significant and clinically relevant differences (mean difference, 4.2 %, 10.2 % and 8.6 %, *P* < 0.001 for all, Bland-Altman statistics) when compared to reference measures (FEV_1_/FEV_6_, FEV_1_/FVC and postbronhodilator FEV_1_/FVC) at tertiary care hospitals with a trend of increasing the difference with lower values (Table 4 and Fig. 3) thus showing a systematic bias.

**Table 4:** Methods comparison (Bland-Altman statistics) for lung function measurements performed in a GP’s office and at lung function lab (*N* = 227)

		COPD-6™ at GP’s office			
Lung function lab measurements			FEV_1_ (L)	FEV_6_ (L)	FEV_1_/FEV_6_ (%)
COPD-6™	FEV_1_ (L)	Δ (95 % CI)	0.01 (−0.05 to 0.03)	NA	NA
	FEV_6_ (L)	Δ (95 % CI)	NA	−0.17 (−0.24 to −0.12)*	NA
	FEV_1_/FEV_6_ (%)	Δ (95 % CI)	NA	NA	4.83 (3.71 to 6.35)*
Spirometry	FEV_1_ (L)	Δ (95 % CI)	−0.12 (−0.15 to −0.09)*	NA	NA
	FVC (L)	Δ (95 % CI)	NA	−0.66 (−0.72 to -0.59)*	NA
	FEV_1_/FVC (%)	Δ (95 % CI)	NA	NA	10.24 (9.32 to 11.26)*
Post-bronchodilator spirometry	FEV_1_ (L)	Δ (95 % CI)	−0.15 (−0.19 to −0.12)*	NA	NA
	FVC (L)	Δ (95 % CI)	NA	−0.60 (−0.65 to −0.54)*	NA
	FEV_1_/FVC (%)	Δ (95 % CI)	NA	NA	8.52 (7.57 to 9.47)*

*GP* general practitioner, *FEV*_*1*_ forced expiratory volume in 1 s, *FEV*_*6*_ forced expiratory volume in 6 s, *FVC* forced expiratory volume, *FVC* forced expiratory volume, Δ mean difference of the index test (COPD-6™ measurement at GP’s office) from the reference (tertiary care measurement), *95 % CI* 95 % confidence interval

*NA* not applicable

*P < 0.001

## Discussion

This study has three main findings: (1) COPD-6™ showed moderate accuracy with high specificity but low sensitivity for COPD; (2) COPD-6™ could be with certain restrictions reliably used in GP’s offices; (3) in the population at risk for COPD, there was a substantial number (18.9 %) of undiagnosed patients. Our data comparing COPD-6™ measurements with a reference standard (tertiary care COPD diagnosis) shows that COPD-6™ can be used with enough accuracy in screening for COPD on a primary care level. Exploratory analyses with the change in threshold showed the possible improvement in the accuracy, but this data needs additional confirmatory studies to be conducted using these values as a pre-specified ones. Although it is our opinion that based on substantial number of undiagnosed patients screening for COPD in a population at risk is valuable, this opinion needs further corroboration based on the studies arguing benefits coming from this effort.

Spirometry is the basis for diagnosing COPD but primary care providers, who first meet patients with respiratory symptoms, do not always have access [15], time or adequate training to use it [16]. In contrast to spirometry, our study showed that COPD-6™ can be reliably used in GP’s offices with the results that are comparable to the measurements performed using the same device by the highly experienced and trained personnel.

Other studies evaluating the use of COPD-6™ differ in many aspects from our study. The population at risk was not strictly defined [7], the patients of older age were included thus increasing the probability of false positive results and inconclusive effects of screening with spirometry [12, 17, 18], and the results of FEV_1_/FEV_6_ measurements were not compared between GP’s offices and lung function laboratory [19]. The results were mainly used as an advanced case finding technique and were not tested for diagnostic purposes. Different cut-off values for FEV_1_/FEV_6_ ratios were suggested with different sensitivity and specificity that didn’t meet the criteria to establish the diagnosis of COPD [20, 21]. Based on the results of our study, the FEV_1_/FEV_6_ ratio <0.7 measured at GP’s office has a specificity of 100 % with positive predictive value of 100 % indicating that a patient had a COPD and can be diagnosed in accordance to other diagnostic criteria with no further lung function testing needed. These results were good but significantly worse than spirometry. The same was the case for the FEV_1_/FEV_6_ ratio measured by COPD-6™ at a lung function lab at tertiary care hospitals using highly experienced staff. The reason behind it, lies in the lack of real-time visual control, present during spirometry, thus underestimating the real value of FEV_6_ (a surrogate measure for FVC). This produced a systematic bias overestimating the real value of FEV_1_/FEV_6_ ratio. This points out that additional training should be provided to heath care personnel at GP’s office to understand and recognize this possible measurement bias.

Otherwise, our exploratory analysis showed that FEV_1_/FEV_6_ ratio >0.85 measured at GP’s office had a NPV of 95.74 % and was reasonable to conclude that a patient was not suffering from COPD and further testing was indicated to reveal the reasons for respiratory symptoms. A strategy of using this two cut-off values (FEV_1_/FEV_6_ < 0.7 to diagnose and >0.85 to rule-out COPD) could increase the number of patients diagnosed at GP’s offices and treated according to the current guidelines. A confirmatory study validating these thresholds should be done using these cut-off values as a pre-specified goals. If this thresholds are confirmed, only patients with the FEV_1_/FEV_6_ ratio between the 0.7 and 0.85 should be referred for further lung function testing and (sub)specialist evaluation. Our results were based on the study population from 40 to 65 years of age so in older populations recommendations from Global Lung Initiative (GLI) to use lower limit of normal (LLN) for FEV_1_/FVC (different from a fixed criterion of <0.7) should be taken into account to prevent over diagnosing COPD in older population [12].

Diagnosing COPD is important because it was shown that undiagnosed patients with COPD have increased mortality [22], morbidity [5, 23] and decreased quality of life [24]. The treatment of such patients is delayed and the probability of quitting smoking is diminished [25]. For a decision to start the implementation of active case finding it is important to know the number of undiagnosed patients in a specific population [26]. There is evidence of different diagnostic accuracy of physician’s established diagnosis for COPD in different countries and age groups [27]. Up till now, we didn’t have a scientific data for our population. In our study, the number of undiagnosed COPD patients in a population at risk is approaching 20 % with almost half of them in advanced stages of the disease (8.4 %). These data are important for health authorities for decision making [28]. Our finding was on a lower end of the results from literature ranging from 20 % to more than 50 % of undiagnosed COPD patients in at risk population [29, 30]. This is probably the result of an overall education campaign started in our country as early as the year 2000 with the presentation of the COPD monograph (COPD guidelines developed by the Croatian Respiratory Society), and followed in subsequent years by the broad education campaign for both GPs and pulmonologists according to GOLD.

**Fig. 4:**
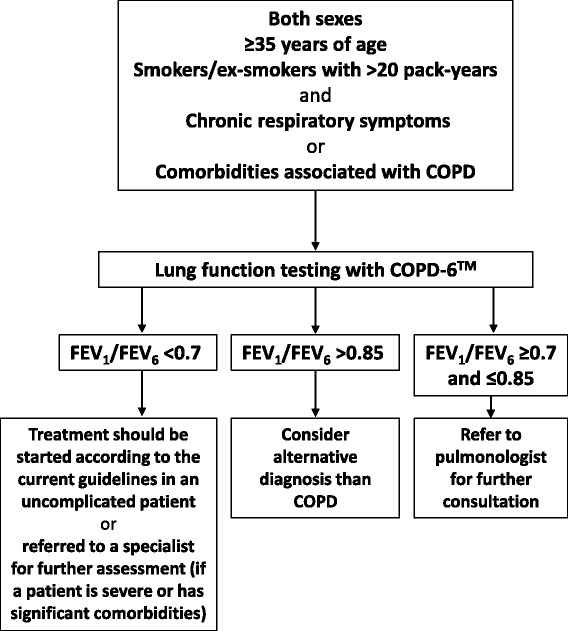
Proposed clinical algorithm for the triage of patient at risk for COPD (smokers/ex-smokers with >20 pack-years having chronic respiratory symptoms or comorbidities associated with COPD) using lung function testing with COPD-6™ at GP’s office

Based on the results of our study we could recommend the clinical algorithm (Fig. 4) for the triage of patient at risk for COPD (smokers/ex-smokers with >20 pack-years having chronic respiratory symptoms or comorbidities associated with COPD) using lung function testing with COPD-6™ at GP’s office (for the age group of >70 cut-off value for the FEV_1_/FEV_6_ should be revised to LLN according to GLI recommendations [12]): (1) FEV_1_/FEV_6_ < 0.7 – treatment should be started according to the current guidelines in an uncomplicated patient or referred to specialist for further assessment if a patient is severe or has significant comorbidities; (2) FEV_1_/FEV_6_ ≥ 0.7 and ≤0.85 – refer to pulmonologist for further consultation; (3) >0.85 should consider alternative diagnosis than COPD. Using such an algorithm in regular GP practice will allow GPs to make an early COPD diagnosis or a proper specialist referral thus producing more appropriate use of resources, cost savings and task shifting. These effects are based on much broader accessibility and lower price of services of general practice and limited resources on the secondary/tertiary care level considering the number of smokers in general population (up to 30 % of adults) and COPD patients (up to 10 % of adults). Substantial positive effects can be expected based on this broad accessibility regarding early diagnosis of COPD allowing early preventive interventions (quitting smoking) [25]. Although it can be supposed that an early intervention already in asymptomatic subjects with fixed airflow limitation could be beneficial there are no studies that confirm such a hypothesis and possible benefits but also no harm could be expected based on studies of therapeutic interventions in mild/moderate COPD [31].

The strengths of our study are based on the significant number of indicative COPD patients diagnosed through the diagnostic process (*n* = 43, 18.9 %), allowing us to properly evaluate the accuracy of the tested device. Also GPs were not extensively trained for the use of the tested device and were not aware to be part of the study thus allowing us to make conclusions that can be generalized to a regular real life clinical setup. Using a “gold standard” to make a COPD diagnosis on the referent level (tertiary care) as a reference standard and evaluation of lung function measurements conducted by GPs against spirometry conducted by experienced staff provides us with an objective evaluation of the tested device and GPs performance. The possible weaknesses of our study are based on recruitment process that may not represent the actual general practice population thus possibly diminishing generalizability of our results, although our analysis showed that there was no clustering present regarding the characteristics of patients recruited by different GPs and the proportion of undiagnosed COPD patients was comparable to other studies. Also the age range of our study participants (40–65 years of age) does not allow us to generalize our results outside this age range thus leaving the most questionable population regarding the diagnostic criteria out of our focus (>70 years of age). A bias could be present because there was no formal panel diagnosis of COPD done, but the diagnosis was done using the harmonized criteria by experienced pulmonologists (all pulmonologist making a diagnosis were acting as trainers for more than 10 years for GOLD initiative in Croatia). So for our data to become more generalizable our research needs to be conducted in a broader population (age 35–80 years, >10 pack-years) using also two threshold values that were found out in our exploratory analyses (FEV_1_/FEV_6_ < 0.7 and >0.85). 

## Conclusions

Almost one fifth (18.9 %) of undiagnosed patients with COPD in a population at risk (smokers/ex-smokers) in our study points to the fact that active case finding should be instituted in such a population. Based on the results of our study lung function testing with COPD-6™ can in a significant part substitute spirometry in cases where it is not readily available to the patient/physician. Results of lung function testing with COPD-6™ performed at GP’s offices and in lung function laboratory were comparable, so there is a possibility to establish the diagnosis of COPD and start adequate treatment in a GP’s office in a substantial number of patients at risk. This could be based on two cut-of values for FEV_1_/FEV_6_, with the ratio <0.7 establishing and >0.85 excluding the diagnosis of COPD. Before the implementation in practice, the diagnostic criteria should be checked for a specific population. Such approach could lead to a better diagnostic yield of COPD in everyday practice diminishing the number of under-diagnosed and over-diagnosed patients.

## Abbreviations

ANOVA: Analysis of variance
ATS: American Thoracic Society
AUC: Area under the curve
COPD: Chronic obstructive pulmonary disease
ERS: European Respiratory Society
FEV_1_: Forced expiratory volume in 1. second
FEV_6_: Forced expiratory volume in 6 s
FVC: Forced vital capacity
GOLD: Global initiative for chronic obstructive lung disease
GP: General practitioner
LLN: Lower limit of normal
NPV: Negative predictive value
PPV: Positive predictive value
QoL: Quality of life
ROC: Receiver operator curve
SD: Standard deviation
